# The unreasonable effectiveness of large language models in zero-shot semantic annotation of legal texts

**DOI:** 10.3389/frai.2023.1279794

**Published:** 2023-11-17

**Authors:** Jaromir Savelka, Kevin D. Ashley

**Affiliations:** ^1^School of Computer Science, Carnegie Mellon University, Pittsburgh, PA, United States; ^2^School of Law, University of Pittsburgh, Pittsburgh, PA, United States

**Keywords:** legal text analytics, large language models (LLM), zero-shot classification, semantic annotation, text annotation

## Abstract

The emergence of ChatGPT has sensitized the general public, including the legal profession, to large language models' (LLMs) potential uses (e.g., document drafting, question answering, and summarization). Although recent studies have shown how well the technology performs in diverse semantic annotation tasks focused on legal texts, an influx of newer, more capable (GPT-4) or cost-effective (GPT-3.5-turbo) models requires another analysis. This paper addresses recent developments in the ability of LLMs to semantically annotate legal texts in zero-shot learning settings. Given the transition to mature generative AI systems, we examine the performance of GPT-4 and GPT-3.5-turbo(-16k), comparing it to the previous generation of GPT models, on three legal text annotation tasks involving diverse documents such as adjudicatory opinions, contractual clauses, or statutory provisions. We also compare the models' performance and cost to better understand the trade-offs. We found that the GPT-4 model clearly outperforms the GPT-3.5 models on two of the three tasks. The cost-effective GPT-3.5-turbo matches the performance of the 20× more expensive text-davinci-003 model. While one can annotate multiple data points within a single prompt, the performance degrades as the size of the batch increases. This work provides valuable information relevant for many practical applications (e.g., in contract review) and research projects (e.g., in empirical legal studies). Legal scholars and practicing lawyers alike can leverage these findings to guide their decisions in integrating LLMs in a wide range of workflows involving semantic annotation of legal texts.

## 1 Introduction

This paper analyzes the capabilities of the newest state-of-the-art generative pre-trained transformers, i.e., GPT-3.5-turbo(-16k) and GPT-4, on semantic annotation of various types of legal texts in zero-shot learning settings. The aim of this paper is to react to the releases and updates of the newest generations of the OpenAI's GPT models and assess if and to what extent the findings presented by similar past studies performed with the text-davinci-003 model still hold. Hence, the focus is not only on the performance of the newest models but also on the comparison of their performance to that of the earlier GPT model. To that end we use selected parts of three legal document corpora that various research groups assembled and manually annotated in the past. We aim to compare the effectiveness of these massively large language models (LLM) in semantically annotating the legal documents. The data sets were carefully selected to represent a wide variety of legal tasks and documents involving semantic annotation. Specifically, we focus on shorter text snippets, usually one or several sentences long, in decisions of the U.S. Board of Veterans' Appeals, commercial contracts, and state statutory provisions dealing with public health emergencies. Hence, we evaluate the effectiveness of the models in tasks that could be part of a typical contract review, statutory/regulatory provisions investigation, or case-law analysis. We also compare the performance of these general (i.e., not fine-tuned^1^) GPT models in annotating small batches of short text snippets coming from such legal documents based on compact, one sentence long, semantic type definitions, against the performance of a traditional statistical machine learning (ML) model (random forest) and fine-tuned BERT model (RoBERTa).

The release of ChatGPT^2^ has made the general public aware of the capabilities of GPT models to write fluent text and answer questions. Although, the underlying GPT-3 technology has been available since 2020 (Brown et al., 2020), the ready availability of this easier-to-use version of the tool is spurring legal practitioners, educators and scholars alike to anticipate how the legal profession will change in the near future or be forced to. Researchers have already applied the technology to tasks traditionally reserved for legal experts. Perlman (2022) claim that ChatGPT requires re-imagining how to obtain legal services and prepare lawyers for their careers. He does so in a human-authored abstract to a scholarly article automatically generated with ChatGPT. Katz et al. (2023) tested GPT-4 on the Uniform Bar Examination (UBE), observing the system comfortably passing the exam. These use cases suggest future applications for the newly emerging technology in providing legal services and increasing access to justice, namely legal document drafting, legal question answering and/or advising, as well as explaining complex legal matters in plain language and summarizing legal documents (Xu and Ashley, 2023).

Semantic annotation of legal texts involves labeling sentences or other portions of legal documents in terms of conceptual types such as rhetorical roles the sentences play in legal arguments, types of contracts clauses, and types or purposes of statutory provisions. While semantic annotation of legal texts may appear less dramatic than these future use cases, we argue that it may prove to be the most promising and valuable application of the massive LLMs to the domain of law. For a variety of reasons (e.g., the danger of misleading advice, professional ethics, limits on who can provide legal services) legal professionals cannot delegate to machines the task of understanding legal documents. Semantic annotation enables conceptually indexing large corpora of legal documents in terms that legal professionals understand and can use to find and review for themselves relevant legal arguments, contractual clauses, or statutory provisions.

Semantic annotation lies at the heart of many high-volume workflows for contract review or case analysis that are currently prohibitively expensive for all but the largest legal teams and projects because they require the time of expensive legal professionals to manually annotate training sets of examples of legal types. Supervised machine learning employs such labeled data in training sets of examples as a step toward learning automatically to annotate previously unseen instances of the conceptual types. GPT models offer the potential for automatic annotation of new instances without any training examples, that is, zero-shot learning. Specifically, the need for manual annotation may be replaced with one or two sentence definitions of the types. The workflow process will become more incremental. Instead of requiring a lot of manual annotation to get started, automation will produce annotations earlier, and legal professionals can focus their time instead on ensuring that the annotations are meaningful and useful. Thus, GPT and similar technology have the potential to (i) dramatically lower the cost of applications such as e-discovery or contract review; (ii) unlock diverse novel applications that would otherwise not be economically feasible; and, perhaps most importantly, (iii) democratize access to these sophisticated workflows that are currently accessible only to a small group of elite legal operatives and their clients.

For example, our work may enable legal practitioners and researchers to undertake efficient exploratory annotation of a dataset of legal texts that does not involve the cost of fine-tuning a large language model. Fine-tuning depends on the availability of labeled data. Often, the process may not require a great deal of labeled data, perhaps only several tens of documents. If one wishes to perform a task on a data set of legal texts that have not yet been labeled, however, the cost can be significant, especially if one is not yet fully certain about which types to include in the type system. The zero-shot approach enables one to sketch and improve the type system incrementally. One lists the types with sentence-long descriptions for each, applies zero-shot annotation with the GPT model, improves the descriptions based on the results, and reapplies the automatic annotation. Depending on the complexity of the task and the intended use of the annotations, the labels produced in this way may often be sufficient. In other cases, they may be a much needed proof of the feasibility of the intended task, making it possible to spend additional resources on human-labeling of a dataset for fine-tuning.

To investigate the capabilities of various state-of-the-art GPT models on semantic annotation of short text snippets from various types of legal documents, we analyzed the following research questions:

1. Using brief type definitions from a non-hierarchical type system describing short snippets of text, how well can GPT-3.5-turbo(-16k) and GPT-4 models classify such texts in terms of the type system's categories as compared to the text-davinci-003 model?
2. What are the effects of performing the annotation on batches of data points compared to annotating each data point individually.

Our work contributes to AI & Law research in the following ways. To our knowledge, this is the first comprehensive study that:

1. Compares the capabilities of text-davinci-003, GPT-3.5-turbo(-16k) and GPT-4 models on semantic annotation of short text snippets from adjudicatory opinions, contractual clauses, and statutory and regulatory provisions in zero-shot settings.
2. Investigates the performance and cost trade-offs of the OpenAI's state-of-the-art GPT models in the context of legal text annotation tasks.

We release the prompts used in our experiments as well as settings for the models in an accompanying GitHub repository.^3^

## 2 Related work

The zero-shot learning capabilities of the GPT models have been recognized by various AI & Law research groups. Yu et al. (2022) explored these in the context of the COLIEE entailment task based on the Japanese Bar exam, improving significantly on the then existing state-of-the-art. Similarly, Katz et al. (2023) successfully applied GPT-4 to the Uniform Bar Examination, and Bommarito et al. (2023) to the Uniform CPA Examination developed by the American Institute of Certified Public Accountants. Sarkar et al. (2021) investigated the potential of various techniques, including LLMs (BERT), in zero/few-shot classification of legal texts. Savelka et al. (2023) employed GPT-4 to evaluate the explanatory value of case sentences that refer to a statutory term of interest. Other studies were focused on the capabilities of the GPT models to conduct legal reasoning (Blair-Stanek et al., 2023; Nguyen et al., 2023), to model U.S. supreme court cases (Hamilton, 2023), to give legal information to laypeople (Tan et al., 2023), and to support online dispute resolution (Westermann et al., 2023).

Analyzing small textual snippets such as sentences (Savelka et al., 2017) in adjudicatory opinions in terms of their function or role is an important task in legal text processing. Prior research utilizing supervised machine learning (ML) or expert crafted rules can roughly be distinguished into two categories. First, the task could be labeling smaller textual units, often sentences, according to some predefined type system (e.g., rhetorical roles, such as evidence, reasoning, conclusion). Examples from several domains and countries include court (Savelka and Ashley, 2017) and administrative decisions from the U.S. (Walker et al., 2019; Zhong et al., 2019), multi-domain court decisions from India (Bhattacharya et al., 2019) or Canada (Xu et al., 2021a,b), international court (Poudyal et al., 2020) or arbitration decisions (Branting et al., 2019), or judicial decisions from multiple countries and legal domains (Savelka et al., 2020). Researchers have also focused on identifying sections that report case outcomes (Petrova et al., 2020; Xu et al., 2020). A second task involves identifying a few contiguous functional parts that comprise multiple paragraphs, as has been done in U.S. (Savelka and Ashley, 2018), Canadian (Farzindar and Lapalme, 2004), French (Boniol et al., 2020), Czech (Harašta et al., 2019), or even multi-jurisdictional (Savelka et al., 2021) legal domains.

Classifying legal norms in terms of their semantic types has been a topic of persistent interest in AI & Law. Researchers have trained traditional statistical supervised ML models on manually annotated texts to identify definitions, prohibitions, or obligations in statutory texts (Biagioli et al., 2005; Francesconi et al., 2010) or to classify sentences in statutes as definitional, publication, or scope of change provisions (de Maat et al., 2010). Other work focuses on finer-grained semantic analysis of statutes to identify obligations, permissions, antecedents, subject agents, or themes (Wyner and Peters, 2011), concepts, and definitions (Winkels and Hoekstra, 2012). A long tradition of analyzing European Union legislative documents also employed manual text annotation (Pouliquen et al., 2006; Boella et al., 2012). ML models trained on sentences from cases that have been manually annotated as better or worse explanations of statutory terms have also learned to select higher quality explanations in new cases (Savelka and Ashley, 2022).

Classification of contractual clauses in terms of various semantic types has also received much interest from the AI & Law community. Chalkidis et al. (2017) employed a combination of statistical ML and hand-crafted rules to analyze the clauses in terms of types such as termination clause, governing law or jurisdiction. Later they utilized various deep learning methods such as CNN, LSTM or BERT (Chalkidis et al., 2021). Leivaditi et al. (2020) made a benchmark data set available comprising 179 lease agreement documents focusing on recognition of entities and red flags, and Wang et al. (2023) released a Merger Agreement Understanding Dataset (MAUD). In our work, we have focused on 12 semantic types from the Contract Understanding Atticus Dataset (CUAD) (Hendrycks et al., 2021).

## 3 Data

As in Savelka (2023), we used three existing manually annotated data sets in our experiments. Each data set supports various tasks involving different types of legal documents. All of them include annotations attached by experts to (usually) short pieces of text. We filtered and processed the data sets to make them suitable for this work's experiments.

### 3.1 BVA decisions of veterans claims

The U.S. Board of Veterans' Appeals^4^ (BVA) is an administrative body within the U.S. Department of Veterans Affairs (VA) responsible for hearing appeals from veterans who are dissatisfied with decisions made by VA regional offices. The BVA reviews a wide range of issues, including claims for disability compensation, survivor benefits, and other compensation and pension claims. Walker et al. (2019) analyzed 50 BVA decisions issued between 2013 and 2017. All of the decisions were arbitrarily selected cases dealing with claims by veterans for service-related post-traumatic stress disorder (PTSD). For each decision, the researchers extracted sentences addressing the factual issues. The sentences were then manually annotated with rhetorical roles they play in the respective decisions (Walker et al., 2017):

- *Finding*—States an authoritative finding, conclusion or determination of the trier of fact—a decision made “as a matter of fact” instead of “as a matter of law.”
- *Reasoning*—Reports the trier of fact's reasoning based on the evidence, or evaluation of the probative value of the evidence, in making the findings of fact.
- *Evidence*—States the content of the testimony of a witness, or the content of documents introduced into evidence, or describes other evidence.
- *Legal Rule*—States one or more legal rules in the abstract, without stating whether the conditions of the rule(s) are satisfied in the case being decided.
- *Citation*—References legal authorities or other materials, and usually contains standard notation that encodes useful information about the cited source.

**Figure 1:**
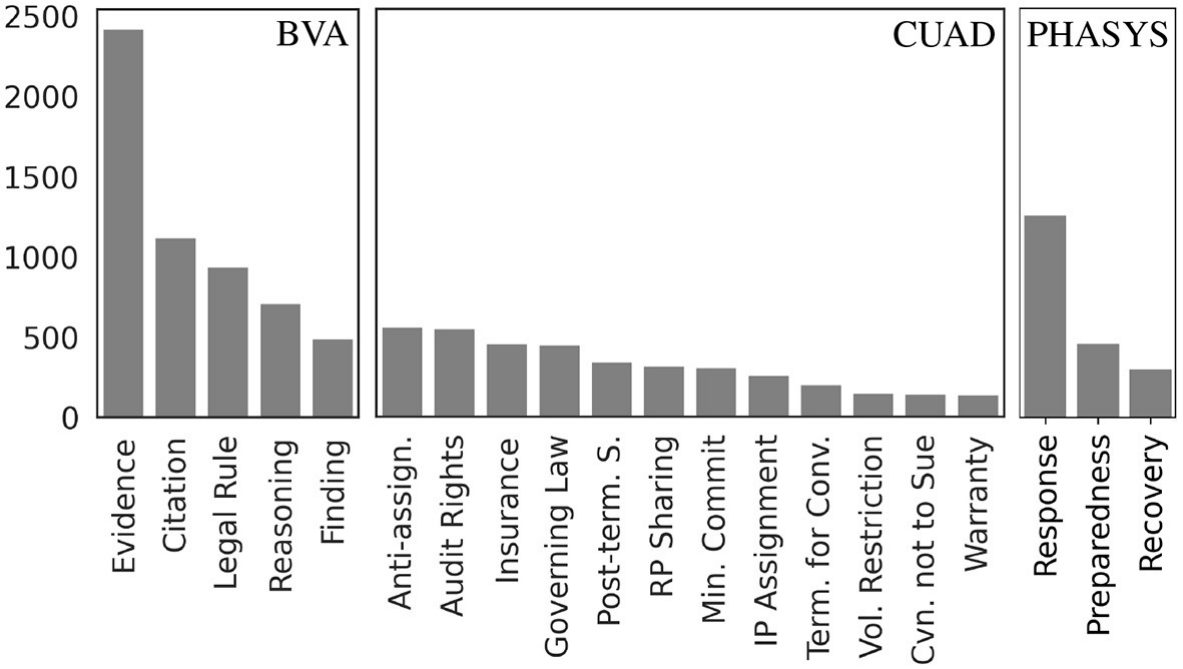
Semantic types distribution. The figure shows the distribution in terms of number of examples of the semantic types across the three data sets. In the BVA data set **(left)**, although *Evidence* is the dominant category, the four remaining types are reasonably represented. The CUAD data set **(center)** does not have a clearly dominant type. Several types are represented by a relatively small number of examples (~100). The PHASYS data set **(right)** is heavily skewed toward the *Response* label (62.4%).

The original PTSD data set^5^ from Walker et al. (2017) contains 478 sentences that are simply annotated as *Sentence*. This is presumably a catch-all category reserved for sentences that do not fit any of the above definitions. Given the nature of our experiments, and since there is no compact definition of this type of sentences provided by the researchers, we exclude sentences of this type from the data set. Figure 1 **(left)** shows the distribution of the labels. While the *Evidence* sentences are clearly the majority label, there is reasonable representation of the other four sentence types. Basic properties of the documents (i.e., sentences from adjudicatory decisions) are provided in Table 1. The sentences are 24.4 words long on average, with the longest sentence having 204 words. We did not perform any other modifications on this data set. Hence, the results reported here can be, with some caveats, compared to the results presented in earlier studies by various groups (Walker et al., 2019; Savelka et al., 2020; Westermann et al., 2021).

**Table 1:** BVA dataset properties.

	**Count**	**Min. len**.	**Mean len**.	**Max. len**.
Finding	485	5	24.4	72
Reasoning	702	3	26.7	92
Evidence	2,416	4	24.8	204
Legal Rule	935	5	34.8	115
Citation	1,112	1	13.5	163
Overall	5,650	1	24.4	204

The figure shows basic properties of the documents, i.e., sentences from adjudicatory decisions, for each of the semantic types. The lengths (min, mean, max) are measured in words.

Sentences in this dataset were classified manually by teams of two trained law students, and they were curated by a law professor with expertise in legal reasoning. The dataset has been released publicly and, hence, open to scrutiny (Walker et al., 2019). While there are detailed publicly-available annotation guidelines related to this data set,^6^ we work with the informal (compact) type definitions provided in the Readme file^7^ of the data set repository in constructing the prompt for the GPT models as in Savelka (2023). These definitions are very close to those provided above.

### 3.2 Contract Understanding Atticus Dataset

The Contract Understanding Atticus Dataset (CUAD) is a corpus of 510 commercial legal contracts that have been manually labeled under the supervision of professional lawyers. This effort resulted in more than 13,000 annotations.^8^ The data set, released by Hendrycks et al. (2021), identifies 41 types of legal clauses that are typically considered important in contract review in connection with corporate transactions. The original 41 categories are a mix of clause-level and sub-sentence-level annotations (e.g., an effective date, names of parties). In this study we work with clause-level types only (typically consisting of one or several sentences). Specifically, we decided to work with the 12 most common clause-level types present in the corpus:

- *Anti-assignment*—Is consent or notice required of a party if the contract is assigned to a third party?
- *Audit Rights*—The right to audit the books, records, or physical locations of the counterparty to ensure compliance.
- *Covenant not to Sue*—Is a party restricted from bringing a claim against the counterparty?
- *Governing Law*—Which state/country's law governs the interpretation of the contract?
- *IP Ownership Assignment*—Does IP created by one party become the property of the counterparty?
- *Insurance*—A requirement for insurance that must be maintained by one party for the benefit of the counterparty.
- *Minimum Commitment*—A minimum amount or units per-time period that one party must buy.
- *Post-termination Services*—Is a party subject to obligations after the termination or expiration of a contract?
- *Revenue-Profit Sharing*—Is one party required to share revenue or profit with the counterparty?
- *Termination for Convenience*—Can a party terminate this contract without cause?
- *Volume Restriction*—A fee increase or consent requirement if one party's use of the product/services exceeds threshold.
- *Warranty Duration*—What is the duration of any warranty against defects or errors?

Figure 1 **(center)** shows the distribution of the clause types. No single type dominates the distribution. The more common types such as *Anti-assignment* or *Audit Rights* each appear more than 500 times, whereas the least represented types such as *Covenant not to Sue* or *Warranty Duration* still have more than 100 examples. Basic properties of the documents (i.e., contractual clauses) are provided in Table 2. The clauses are 48.7 words long on average, with the longest sentence having 703 words. Besides the above described filtering, we did not perform any other transformations on this data set. Hence, we focus on a subset of tasks described in Hendrycks et al. (2021).

**Table 2:** CUAD dataset properties.

	**Count**	**Min. len**.	**Mean len**.	**Max. len**.
Anti-assignment	549	5	46.1	703
Audit rights	540	8	46.6	219
Covenant not to Sue	136	12	65.8	340
Governing law	440	7	33.8	141
IP ownership assignment	252	6	61.5	217
Insurance	446	5	44.1	240
Minimum commitment	300	2	51.4	306
Post-termination services	336	14	70.2	275
Revenue-profit sharing	311	10	49.7	210
Termination for convenience	195	4	38.4	333
Volume restriction	143	12	43.6	164
Warranty duration	135	10	48.3	145
Overall	3,783	2	48.7	703

The figure shows basic properties of the documents, i.e., contractual clauses, for each of the semantic types. The lengths (min, mean, max) are measured in words.

Hendrycks et al. (2021) states that the contracts were labeled by law students and checked by experienced lawyers. The law students went through 70–100 hours of training for labeling that was designed by experienced lawyers. The process was supported by extensive documentation on how to identify each label category in a contract that takes up more than one hundred pages. The data set includes brief, one sentence long, type definitions that are publicly available.^9^ These definitions roughly correspond to those provided above. We utilize these definitions in the GPT models' prompt construction (see Section 4.2 for details).

### 3.3 PHASYS statutes and regulations analysis

At the University of Pittsburgh's Graduate School of Public Health, researchers have manually coded state and local laws and regulations related to emergency preparedness and response of the public health system (PHS). They use the codes to generate, analyze and compare network diagrams representing various functional features of states' regulatory frameworks for public health emergency preparedness. As described more fully in Sweeney et al. (2013), they retrieved candidate sets of relevant statutes and regulations from a full-text legal information service and identified relevant spans of text. They then coded the relevant spans as per instructions in the codebook,^10^ representing relevant features of those spans as sequences of numerical codes. In this work we focus on one specific dimension of that code, namely the purpose of the legal provision in terms of the following three categories:

- *Emergency Preparedness*—An effort to plan for a disaster/emergency before it occurs (also “emergency readiness”).
- *Emergency Response*—An effort to lessen the impact of a disaster/emergency after it occurs.
- *Emergency Recovery*—An effort to respond to the impact of a disaster/emergency after it has ended in an attempt to return to the state of normalcy.

Categorizing states' emergency-related statutory provisions by purpose facilitated cross-state comparisons.

Following the approach described in Savelka et al. (2014), the statutory and regulatory texts were automatically divided into text units which are often non-contiguous spans of text which may be referenced with citations. A citation is understood as a unique path through a tree representing a structure of a document. Each text unit contains pieces of texts that can be found at each node of such path.

Figure 1 **(right)** shows the distribution of the provision types according to their purpose. The *Response* category is clearly dominating the distribution with 1,231 occurrences (62.4%). This problematic class imbalance creates interesting issues and questions that warrant further investigations (Section 6). Basic properties of the documents (i.e., statutory provisions) are provided in Table 3. The provisions are 140.4 words long on average, with the longest sentence having 6,390 words. The codebook mentioned above provides short (i.e., one sentence long) definitions for each of the three types. These definitions roughly correspond to those provided above. We worked with these definitions in constructing the prompt for the GPT models (Section 4.2).

**Table 3:** PHASYS dataset properties.

	**Count**	**Min. len**.	**Mean len**.	**Max. len**.
Emergency preparedness	450	22	110.9	1,062
Emergency response	1,231	22	145.7	6,390
Emergency recovery	291	29	164.1	3,459
Overall	1,972	22	140.4	6,390

The figure shows basic properties of the documents, i.e., statutory provisions, for each of the semantic types. The lengths (min, mean, max) are measured in words.

## 4 Experiments

We used several systems and experimental setups as baselines to the GPT models applied in the zero-shot settings. We describe the models used as baselines, the evaluated GPT models, and the experimental settings in the subsections below.

### 4.1 Models

#### 4.1.1 Random forest

A random forest (Ho, 1995) is an ensemble classifier that fits a number of decision trees on sub-samples of the data set. It can be understood as a team of experts (the decision trees) each examining different pieces of the data (sub-samples). After all experts have analyzed their pieces, they come together and make a final decision through averaging. This approach helps to not only improve the predictive accuracy but also to prevent overfitting—a common pitfall where a model performs well on the training data but fails to generalize to unseen data. As an implementation of random forest we used the scikit-learn's Random Forest Classifier module.^11^

Including random forest in our experiments serves to compare the GPT models to a well-regarded traditional ML technique. Note that the random forest model still does not capture semantics as more advanced models do. Also note, that random forest as a supervised ML model requires training data which is in contrast to the evaluated GPT models that do not require any task specific training data in the zero-shot settings.

#### 4.1.2 RoBERTa

BERT (bidirectional encoder representation from transformers) (Devlin et al., 2018), based on the transformer architecture from Vaswani et al. (2017), has gained immense popularity. A large number of models using similar architectures have been proposed, e.g., RoBERTa (Liu et al., 2019), ALBERT (Lan et al., 2019), or T5 (Raffel et al., 2019). The core capability of these models is their fine-tuning on a downstream task. The original model is typically trained on large corpora of general language resources, such as Wikipedia or book corpora, to perform weakly supervised tasks such as masked token prediction or the next sentence prediction. For a downstream task one typically adds to the core model a small layer that handles, e.g., the classification into specific classes, such as in this work. Using a task specific data set, the augmented model is then further trained (fine-tuned) starting from the parameters optimized during the pre-training phase.

In this work, we use RoBERTa (a robustly optimized BERT pre-training approach) described in Liu et al. (2019).^12^ Out of the available models, we chose to work with the smaller roberta-base model that has 125 million parameters. RoBERTa is using the same architecture as BERT. However, the authors of Liu et al. (2019) conducted a replication study of BERT pre-training and found that BERT was significantly undertrained. They used the insights thus gained to propose a better pre-training procedure. Their modifications include longer training with bigger batches and more data, removal of the next sentence prediction objective, training on longer sequences on average (still limited to 512 tokens), and dynamic changing of the masking pattern applied to the training data (Liu et al., 2019). Note that there are other models that would presumably perform better than the roberta-base model used in this work. For example, the larger models would likely achieve better performance. Additionally, there are models that have been pre-trained on legal texts such as those presented in Chalkidis et al. (2020) or Zheng et al. (2021). However, this paper is not about matching or out-performing the state-of-the-art. This paper is about showing that the modern GPT models can perform reasonably well in zero-shot settings. Hence, the widely used fine-tuned roberta-base is used as an upper-bound baseline. Note that the fine-tuning step requires task specific annotated data.

#### 4.1.3 Generative pre-trained transformers (GPT)

The original GPT model (Radford et al., 2018) is a 12-layer decoder-only transformer with masked self-attention heads. Its core capability is likewise the fine-tuning on a downstream task. The GPT-2 model (Radford et al., 2019) largely follows the details of the original GPT model with a few modifications, such as layer normalization moved to the input of each sub-block, additional layer-normalization after the first self-attention block, and a modified initialization. Compared to the original model it displays remarkable multi-task learning capabilities (Radford et al., 2019). The next generation of GPT models (Brown et al., 2020) uses almost the same architecture as GPT-2. The only difference is that it uses alternating dense and locally banded sparse attention patterns in the layers of the transformer. The main focus of Brown et al. (2020) was to study the dependence of performance on model size where eight differently sized models were trained (from 125 million to 175 billion parameters). The largest of the models is commonly referred to as GPT-3. The interesting property of these models is that they appear to be very strong zero- and few-shot learners. This ability appears to improve with the increasing size of the model (Brown et al., 2020). The technical details about the recently released GPT-4 model have not been disclosed due to concerns about potential misuses of the technology as well as a highly competitive market for generative AI (OpenAI, 2023).

In our experiments, we used gpt-4 (GPT-4), gpt-3.5-turbo(-16k) and text-davinci-003 (both GPT-3.5). As of this writing, GPT-4 is by far the most advanced model released by OpenAI. The GPT-4 and gpt-3.5-turbo(-16k) models are focused on dialog between a user and a system. On the other hand, text-davinci-003 is a more general model focused on text completion. It builds on the previous text-davinci-002 (Ouyang et al., 2022). That, in turn, is based on code-davinci-002. It is focused on code-completion tasks and is sometimes referred to as codex (Chen et al., 2021).^13^

### 4.2 Experimental design

#### 4.2.1 Baselines

For the random forest classifier, we split each of the three data sets into 10 similarly sized folds. The split was performed at the level of documents in which the evaluated text snippets were contained. As a result all the text snippets from a particular document were assigned to the same fold (e.g., all the sentences from a single BVA decision). This was important to safe-guard against pieces of text from a single document being included in the training set as well as the test set. Within each iteration of the cross-validation, we utilized grid search.^14^ to select the best set of hyperparameters (5-fold internal cross-validation on the training set). The hyperparameter space that was being considered was defined over the type of n-grams to be used (1-grams, {1 2}-grams, or {1 2,3}-grams), number of estimators (10, 100) and maximum tree depth (8, unlimited).

We fine-tuned the base RoBERTa model for 10 epochs on the training set within each of the cross-validation folds.^15^ We employed the same splits as in evaluating the random forest's performance. We set the sequence length to 512 and the batch size to 16. As optimizer we employed the Adam algorithm (Kingma and Ba, 2014) with initial learning rate set to 4*e*^−5^. We stored a model's checkpoint after the end of each training epoch. The best performing checkpoint evaluated on the training set was used to make the predictions on the corresponding test fold.

#### 4.2.2 GPT

In evaluating the performance of the general (not fine-tuned) GPT models, we applied them to a batch of text snippets using the openai Python library^16^ which is a wrapper for the OpenAI's REST API.^17^ In an attempt to minimize costs, we made the batches as large as possible. Their size is limited by the size of the evaluated text snippets that can fit into the prompt (8k tokens for GPT-4, 4k tokens for gpt-3.5-turbo and text-davinci-003, and 16k for gpt-3.5-turbo-16k), while still leaving enough room for the completed predictions. For text-davinci-003 we re-use the baseline experiments performed in Savelka (2023) where the batch sizes were set to 50 for the BVA decision sentences, 20 for the CUAD contractual clauses, and 10 for the PHASYS statutory and regulatory provisions. In this work we use dynamically sized batches for the GPT-4 and gpt-3.5-turbo(-16k) fitting as many data points into a batch as the above described prompt limitations allow.

**Figure 2:**
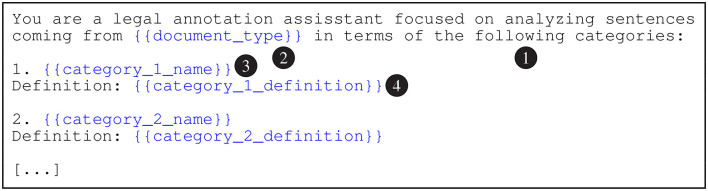
The system prompt template for GPT-4 and gpt-3.5-turbo(-16k) models. The preamble (1) primes the model to generate semantic type predictions. The document_type token (2) is replaced with the document type according to the data set (e.g., “adjudicatory decisions” for BVA). The category_n_name tokens (3) are substituted with the names of the semantic types and the category_n_definition tokens (4) with the corresponding definitions.

**Figure 3:**
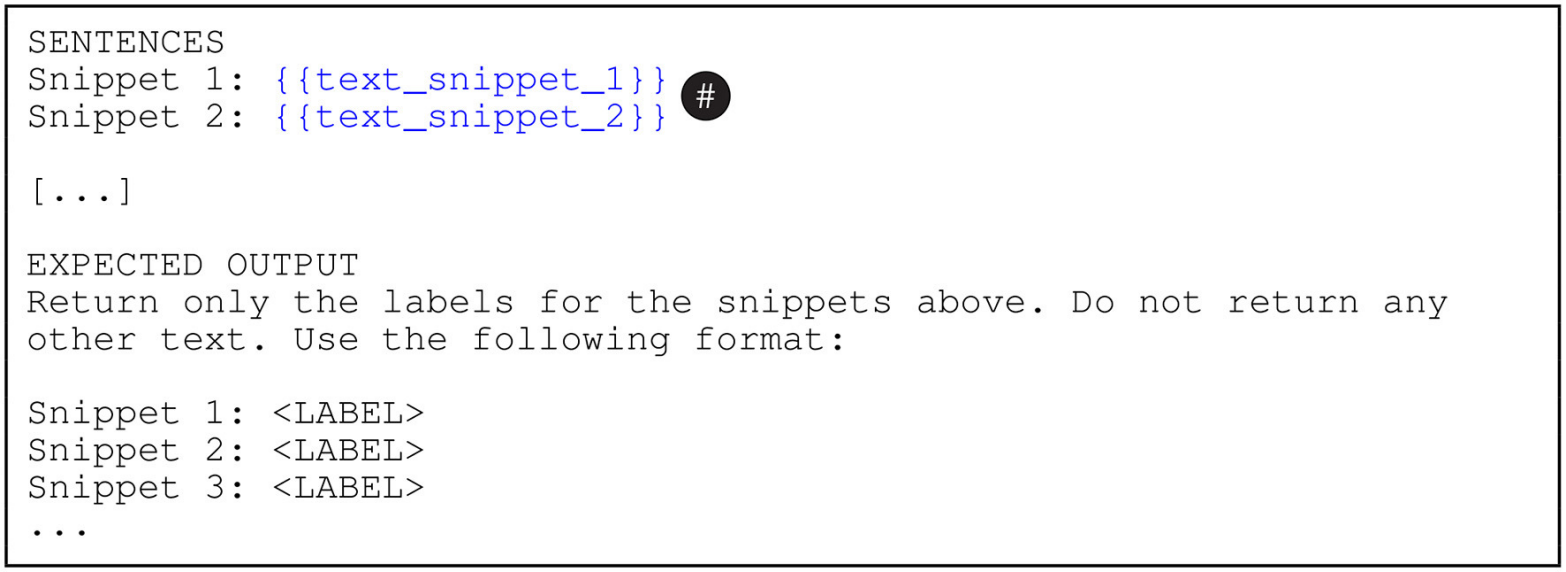
The user prompt template for the GPT-4 and gpt-3.5-turbo(-16k) models. The text_snippet_n tokens (#) are replaced with the analyzed text snippets.

**Figure 4:**
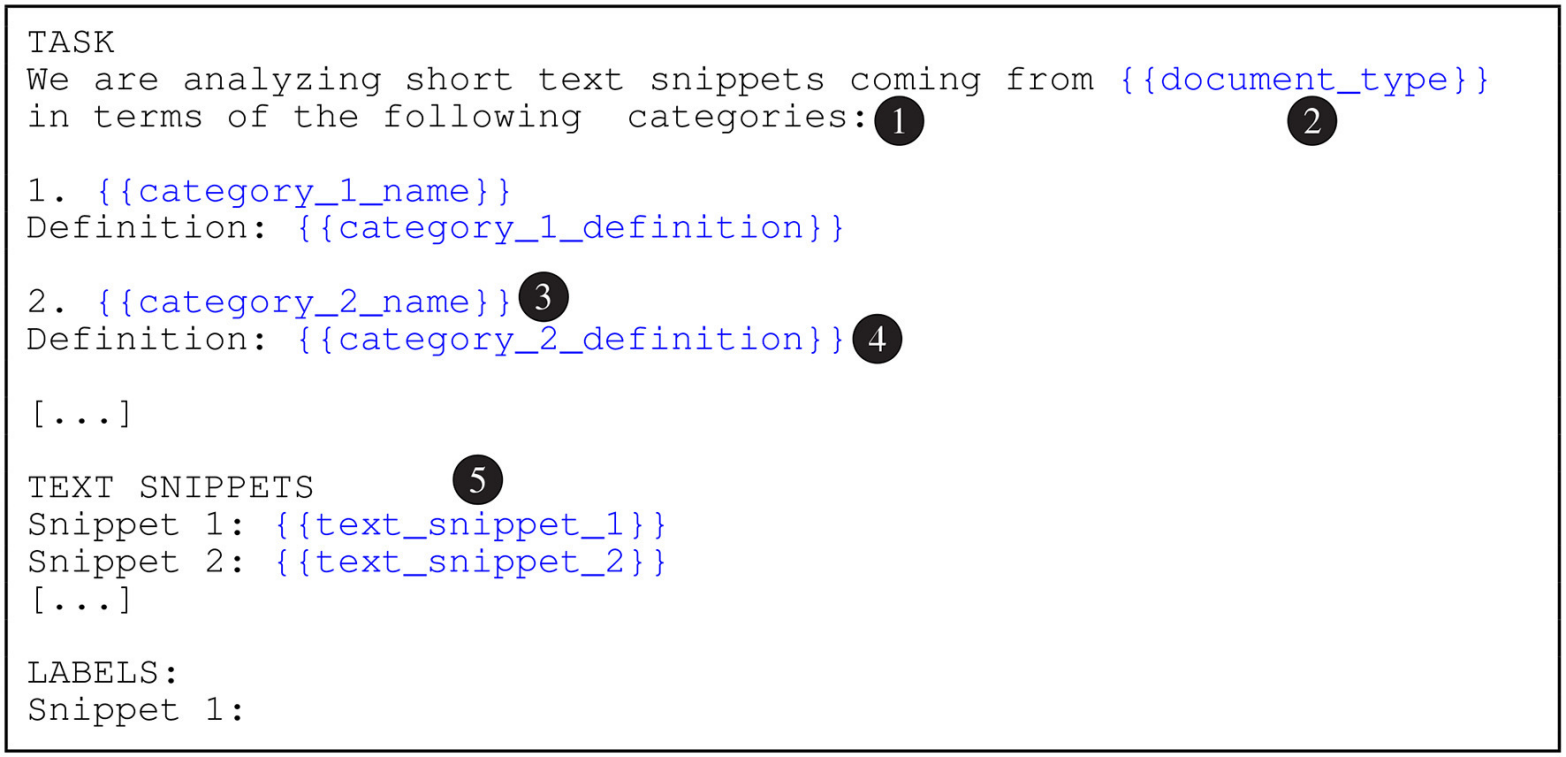
The prompt template for text-davinci-003 Model. The preamble (1) primes the model to generate semantic type predictions. The document_type token (2) is replaced with the document type according to the data set (e.g., “adjudicatory decisions” for BVA). The category_n_name tokens (3) are substituted with the names of the semantic types and the category_n_definition tokens (4) with the corresponding definitions. The text_snippet_n tokens (5) are replaced with the analyzed text snippets.

To generate the automatic predictions, we embed each batch in the prompt templates shown in Figures 2–4. In these figures, the {{document_type}} tokens are replaced with “adjudicatory decisions” (BVA), “contracts” (CUAD), or “statutes and regulations” (PHASYS). We replace the {{category_n_name}} tokens with the names of the semantic types from the type systems and the {{category_n_definition}} tokens with their corresponding definitions. Finally, the tokens marked as {{text_snippet_n}} are replaced with the analyzed text snippets. The models return the list of predicted labels as the response (prompt completion). We emphasize that the construction of the prompts is focused on maximizing the cost effectiveness, therefore accessibility, of the proposed approach which may somewhat limit the performance of the evaluated GPT models. This important issue is further discussed in Section 6.

We set the GPT parameters as follows. Temperature controls the creativeness of the output: the higher the temperature the more creative the output but it can also be less factual. We set it to 0.0, which corresponds to no randomness. The top_p parameter is related to temperature and also influences creativeness of the output. We set top_p to 1, as is recommended when temperature is set to 0.0. The max_tokens parameter controls the maximum length of the output. We set it to 500 (a token roughly corresponds to a word). The frequency_penalty parameter affects repetitiveness. We set it to 0, which allows repetition by ensuring no penalty is applied to repetitions. Finally, we set the related parameter, presence_penalty, to 0, ensuring no penalty is applied to tokens appearing multiple times in the output, which happens frequently in our use case.

#### 4.2.3 Evaluation measures

We use precision (*P*), recall (*R*), and F_1_-measure (F_1_), i.e., the traditional information retrieval measures, to evaluate performance of the various systems.^18^ The performance is evaluated at the level of the individual text snippets for each semantic type. Therefore, *P*^*j*^, *R*^*j*^, and $F_{1}^{j}$ for a semantic type *T*_*j*_ are computed as follows:


$$\begin{array}{lll} P^{j} & = & \overset{\left|S\right|}{\underset{i=1}{\sum }}\frac{t_{h}\left(s_{i}\right)=T_{j}\wedge t_{a}\left(s_{i}\right)=T_{j}}{t_{a}\left(s_{i}\right)=T_{j}}\\ R^{j} & = & \overset{\left|S\right|}{\underset{i=1}{\sum }}\frac{t_{h}\left(s_{i}\right)=T_{j}\wedge t_{a}\left(s_{i}\right)=T_{j}}{t_{h}\left(s_{i}\right)=T_{j}}\\ F_{1}^{j} & = & \frac{2P^{j}R^{j}}{P^{j}+R^{j}} \end{array}$$

*S* stands for the set of all text snippets in a data set (e.g., all sentences from BVA decisions); *T*_*j*_ represent a specific type (e.g., *Finding*); *t*_*h*_(*s*_*i*_) stands for a human expert annotation of sentence *s*_*i*_; and *t*_*a*_(*s*_*i*_) is an annotation of sentence *s*_*i*_ generated automatically. The overall *P*, *R*, and *F*_1_ measures for each data set are computed at the micro level.

## 5 Results

The experimental results of applying the GPT and the baseline models to the three tasks involving adjudicatory opinions (BVA), contract clauses (CUAD) and statutory and regulatory provisions (PHASYS) are shown in Table 4. It appears that the zero-shot GPT models perform reasonably well. This is especially apparent when one considers that they operate solely on the basis of the lists of the types with one sentence long descriptions. The zero-shot models are even quite competitive with the supervised ML algorithms trained on the in-domain data. The supervised models have been trained on large portions of the available data (i.e., several thousand examples). The GPT models have not been trained on any in-domain annotated examples. The GPT-4 model even matches the performance of the random forest model on the BVA and CUAD tasks. It does not match the performance of the fine-tuned RoBERTa model, which is to be expected. It is important to appreciate that the RoBERTa model utilized thousands of task-specific annotated data to reach the reported performance whereas the GPT-4 model did not have access to any such data. The performance of the gpt-3.5-turbo(-16k) models is somewhat lower as compared to the GPT-4 model. This is not surprising as GPT-4 is a much more powerful as well as more expensive model. Interestingly, the cost-effective gpt-3.5-turbo model performs comparably to the much more expensive text-davinci-003. The lower performance of the gpt-3.5-turbo-16k is most likely due to the large size of the prompt where too many data points might have been included in a single batch.

**Table 4:** Experimental results.

	**RandF**			**BERT**			**td-003**			**gpt-3.5**			**gpt-16k**			**gpt-4**		
	**P**	**R**	**F**_1_	**P**	**R**	**F**_1_	**P**	**R**	**F**_1_	**P**	**R**	**F**_1_	**P**	**R**	**F**_1_	**P**	**R**	**F**_1_
**BVA**	**0.84**	**0.84**	**0.83**	**0.92**	**0.92**	**0.92**	**0.81**	**0.70**	**0.73**	**0.80**	**0.68**	**0.71**	**0.73**	**0.53**	**0.57**	**0.84**	**0.81**	**0.82**
Citation	0.99	0.98	0.99	1.0	1.0	1.0	0.98	0.94	0.96	0.95	0.90	0.92	0.86	0.40	0.55	0.97	0.88	0.92
Evidence	0.79	0.98	0.87	0.94	0.95	0.94	0.93	0.65	0.77	0.92	0.64	0.75	0.92	0.50	0.64	0.92	0.84	0.88
Finding	0.82	0.66	0.74	0.85	0.88	0.87	0.50	0.56	0.53	0.56	0.59	0.57	0.61	0.39	0.47	0.66	0.73	0.70
Legal rule	0.92	0.85	0.89	0.94	0.96	0.95	0.86	0.61	0.72	0.82	0.52	0.64	0.53	0.64	0.58	0.85	0.84	0.84
Reasoning	0.70	0.27	0.40	0.74	0.70	0.72	0.30	0.72	0.43	0.29	0.76	0.42	0.24	0.82	0.37	0.44	0.63	0.52
**CUAD**	**0.89**	**0.89**	**0.89**	**0.95**	**0.95**	**0.95**	**0.84**	**0.84**	**0.83**	**0.87**	**0.86**	**0.86**	**0.81**	**0.80**	**0.80**	**0.90**	**0.90**	**0.90**
Anti-assign0.	0.91	0.97	0.94	0.99	0.98	0.99	0.81	0.94	0.87	0.93	0.93	0.93	0.92	0.88	0.90	0.92	0.95	0.93
Audit rights	0.86	0.96	0.91	0.96	0.97	0.97	0.95	0.91	0.93	0.96	0.89	0.93	0.89	0.82	0.86	0.94	0.96	0.95
C0. not to sue	0.97	0.81	0.88	0.97	0.94	0.96	0.73	0.71	0.72	0.77	0.83	0.80	0.65	0.81	0.72	0.94	0.91	0.93
Governing law	1.0	1.0	1.0	0.99	1.0	1.0	0.99	1.0	0.99	1.0	1.0	1.0	0.99	0.94	0.96	0.98	0.98	0.98
IP assignment	0.90	0.86	0.88	0.94	0.93	0.93	0.75	0.96	0.84	0.71	0.96	0.81	0.63	0.89	0.74	0.90	0.91	0.91
Insurance	0.94	0.97	0.95	0.97	0.97	0.97	0.97	0.95	0.96	0.98	0.95	0.97	0.96	0.87	0.92	0.96	0.98	0.97
Min. commit.	0.82	0.79	0.80	0.92	0.93	0.92	0.68	0.67	0.67	0.82	0.66	0.73	0.71	0.60	0.65	0.82	0.79	0.80
Post-term. S.	0.78	0.76	0.77	0.85	0.85	0.85	0.80	0.42	0.55	0.64	0.78	0.70	0.55	0.70	0.62	0.81	0.79	0.80
Profit sharing	0.82	0.92	0.87	0.94	0.94	0.94	0.76	0.81	0.78	0.88	0.87	0.87	0.77	0.81	0.79	0.91	0.89	0.90
Termination C.	0.90	0.88	0.89	0.95	0.96	0.96	0.83	0.96	0.89	0.85	0.93	0.89	0.80	0.84	0.82	0.86	0.97	0.91
Volume rest.	0.86	0.50	0.63	0.90	0.90	0.90	0.47	0.45	0.46	0.47	0.29	0.36	0.49	0.27	0.35	0.64	0.48	0.55
Warranty dur.	0.95	0.79	0.86	0.95	0.93	0.94	0.82	0.81	0.81	0.91	0.74	0.82	0.80	0.70	0.75	0.92	0.89	0.91
**PHASYS**	**0.69**	**0.69**	**0.64**	**0.74**	**0.75**	**0.74**	**0.64**	**0.54**	**0.54**	**0.68**	**0.51**	**0.53**	**0.68**	**0.31**	**0.24**	**0.67**	**0.53**	**0.54**
Response	0.69	0.95	0.80	0.80	0.84	0.82	0.72	0.57	0.63	0.79	0.44	0.56	0.83	0.11	0.20	0.78	0.43	0.55
Preparedness	0.63	0.18	0.28	0.64	0.56	0.60	0.33	0.68	0.45	0.32	0.83	0.46	0.25	0.97	0.39	0.33	0.82	0.47
Recovery	0.82	0.40	0.53	0.65	0.63	0.64	0.77	0.17	0.28	0.77	0.36	0.49	0.71	0.09	0.16	0.76	0.49	0.60

The performance is reported in terms of F_1_ scores. The micro-P, R, and F_1_ are used for the overall data set statistics (BVA, CUAD, PHASYS rows). RandF means random forest and BERT means base RoBERTa. The td-003 section reports the performance of the text-davinci-003 model. The gpt-3.5 and gpt-16k refer to the performance of the gpt-3.5-turbo(-16k) models, respectively. The gpt-4 column reports the performance of the most powerful GPT-4 model. The bold values describe the overall performance of the models on the datasets.

Table 5's three confusion matrices provide more detailed information about the performance of the GPT-4 model under the zero-shot condition. Regarding the CUAD contractual clauses, the system appears to have problems distinguishing only a small number of classes, such as *Minimum Commitment, Profit Sharing*, or *Volume Restrictions*. As for the BVA adjudicatory decisions, the *Reasoning* class appears to be the most problematic. The system misclassifies a large number of *Evidence* sentences (654) as *Reasoning*. The PHASYS statutory and regulatory provisions seem to present the greatest challenge. The system mislabels a large number of *Emergency Response* provisions as *Emergency Preparedness*.

**Table 5:** Confusion matrices of the GPT-4 model under the zero-shot learning condition.

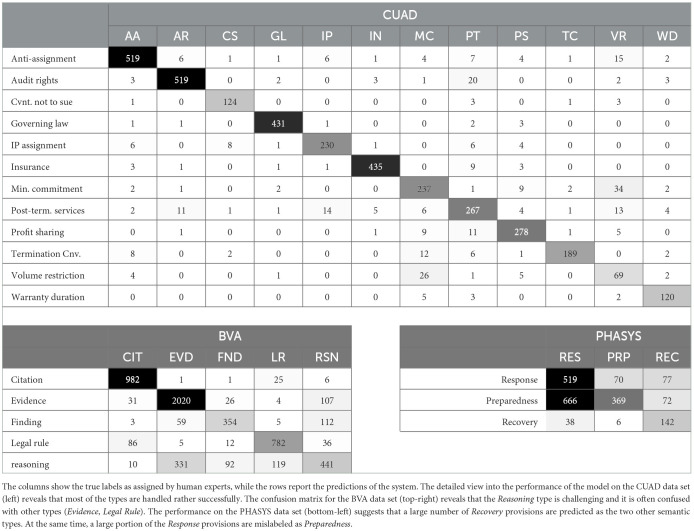

## 6 Discussion

The performance of the GPT models in the zero-shot settings suggests the approach's promising potential in semantically annotating short text snippets coming from legal documents. The results indicate the feasibility of the zero-shot approach in many existing workflows that rely on semantic annotation (e.g., in contract review or case-law analysis). Various applications of the approach (e.g., standalone, human-in-the-loop) are likely to dramatically lower the cost of experimenting with and performing such workflows. The technical barriers for operating the GPT models are relatively low—none in case of some variants such as ChatGPT. The economic barriers are also (almost) non-existent when it comes to experimenting and relatively low when it comes to running the workflows on document sets of typical sizes (e.g., hundreds or lower thousands of contracts) when the batch prediction (as employed here) is utilized.

In this study, we consider the cost of the proposed approach as an important factor. This is because the most valuable benefit of zero-shot LLMs could be the democratization of access to the sophisticated workflows that involve semantic annotation of legal documents. Such workflows are currently accessible to only a small group of elite legal operations due to their requirements on technical expertise and workload scale. Performing semantic annotation via a zero-shot LLM will be far less expensive than manual annotation or the use of existing enterprise solutions based on the supervised ML paradigm. Nevertheless, it may still incur non-negligible cost.

For the sake of the analysis here, let us consider the current pricing scheme of text-davinci-003 [cheaper than GPT-4 but more expensive than gpt-3.5-turbo(-16k) models] based on the number of tokens submitted to and generated by the model. Currently, the cost is set to $0.02 per 1,000 tokens in the prompt, including the completion.^19^ Assuming we would like to maximize the model performance, disregarding the cost, we could analyze each data point (i.e., text snippet) individually utilizing the available prompt to its full potential. This could entail providing more verbose definitions of the semantic types, as well as supplying larger number of examples. While this could lead to a better performance in the task of analyzing the text snippets, the number of exchanged tokens would rise dramatically across the three tasks presented in this study.

The task of analyzing 50 adjudicatory decisions (such as in the BVA corpus) is fairly realistic despite the fact that, in practice, much larger number of documents may be involved. Using the batch approach employed in this study, the cost of such analysis would amount to no more than $9.26.^20^ Maximizing the performance and analyzing one data point per prompt, on the other hand, could cost $462.96. For CUAD the discrepancy would be $15.50 (batch) compared to $309.98 (one data point) and for PHASYS it would be $16.16 (batch) vs. $161.59 (one data point). Hence, assuming comparable numbers of documents to those present in the data sets studied in this work, the batch approach incurs costs in the ballpark of several to at most the lower tens of dollars. The approach analyzing one text snippet at a time could amount to the cost of several hundred dollars. While such a cost may still be considered acceptable in many use cases, it presents a significant barrier in many others.

After exploring the potential cost differences between the approaches, we would also like to understand the differences in performance. To that end we conducted a limited experiment on the BVA data set that benchmarks (i) the batch approach used in this study to (ii) the approach where the prompt is kept exactly the same except only one example at a time being submitted (as opposed to a batch of 50). The limited experiment was performed on 10 randomly sampled BVA decisions (1,175 sentences overall).

Table 6 reports the results of the above experiment. First of all, it appears clear that focusing the model on predicting just one single label as opposed to a batch of 50 labels somewhat improves the performance. It is an open question whether the increase of F_1_ from 0.80 to 0.81 justifies 12.6 times higher cost. The answer likely depends on particular circumstances. While the absolute monetary figures listed in Table 6 might not appear prohibitive, a realistic analysis would likely involve a greater number of opinions than the 10 included in this limited experiment. There, the difference in cost may determine whether the analysis is economically viable or prohibitively expensive. Note that including an excessive number of examples in a single prompt may seriously degrade the performance as evidenced by the performance of the gpt-3-5-turbo-16k (Table 4).

**Table 6:** The effects of prediction batching.

	**P**	**R**	**F_1_**	**Tokens**	**Cost**
Batches of 50 data points	0.85	0.78	0.80	71,600	$1.43
One per prompt	0.87	0.79	0.81	897,751	$17.96

The table shows performance of the text-davinci-003 model in terms of Precision (P), Recall (R) and micro F_1_ measure (F_1_). The Tokens column reports the number of tokens each approach exchanged with the model. The Cost column estimates the cost of such exchange given the OpenAI's pricing scheme for the text-davinci-003 model as of the publication of this paper.

Perhaps the most exciting application of the zero-shot semantic analysis could be the enabling of unique workflows that would have traditionally been performed manually or deemed not feasible. It is often the case that the cost of manual annotation performed by a human with rare and valuable expertise is not economically viable. Authoring of precise and verbose annotation guidelines as well as training of other individuals to perform the task according to the guidelines might be equally expensive. Hence, an approach is appealing that requires no more than specifying compact (one sentence long) semantic type definitions. The effectiveness of an LLM utilizing such definitions could be evaluated, at least in a limited way, by (i) applying the model to no more than a few hundred data points, and (ii) an expert manually inspecting the predictions. The appeal of this approach extends equally to legal scholarship, especially empirical legal studies (see, e.g., Gray et al., 2023). We (optimistically) anticipate that large scale expensive projects of the past may become routine endeavors in the near future.

## 7 Limitations

While the results of our experiments are promising, limitations clearly exist. First, the performance of the models is far from perfect and there is still a gap between the performance of the zero-shot LLM compared to the performance of the fine-tuned LLM systems trained on thousands of example data points. Hence, in workflows with low tolerance for inaccuracies in semantic annotation, the zero-shot LLM predictions may require keeping a human-expert in the loop. The outcome of such human-computer interaction may be a high quality data set of the size that enables fine-tuning of a powerful domain-adapted LLM.

The performance of the zero-shot approach differs across the three data sets. While the performance on the CUAD data set seems very reasonable, the performance on the BVA data set suffers from some clear limitations. Specifically, the model struggles with the *Reasoning* type. It mislabels many sentences of other types as *Reasoning* and fails to recognize many *Reasoning* sentences as such (Table 5). This is consistent with the performance of the supervised ML models. While the fine-tuned base RoBERTa is clearly more successful in handling this semantic type compared to the zero-shot GPT models, it still struggles (F_1_ = 0.71). The random forest model under-performs the zero-shot models. Hence, the correct recognition of this type may require extremely nuanced notions that may be difficult to acquire through a compact one-sentence definition (zero-shot GPT models) or word occurrence features (random forest). For such situations, the proposed approach might not (yet) be powerful enough and the only viable solution could be fine-tuning an LLM.

The performance of the zero-shot GPT models on the PHASYS data set is not very satisfactory and warrants further investigation. For the purpose of this study, we identify several challenges this data set poses that make it difficult even for the supervised ML models (Table 4). First of all, the data set is clearly imbalanced with the *Response* semantic type constituting 62.4% of the available data points. Second, the definitions of the semantic types appear to be somewhat less clear and of lower quality than for the other two data sets (Section 3.3). The PHASYS annotation guidelines often list just the names of the types and do not even include definitions. Hence, we hypothesize that the manual annotation of this data set heavily relied on the informal expertise of the human annotators, which was not adequately captured in the annotation guidelines. Finally, there might be the same issue as with the *Reasoning* type from the BVA data set. The fine-grained distinctions between what counts as emergency *Response* as opposed to *Preparedness* may simply be too nuanced to be captured in a compact definition.

A related limitation stems from our focus on annotation tasks involving relatively brief type definitions from non-hierarchical type systems describing relatively short snippets of text. Various legal domain tasks need to be performed on longer snippets of text that involve more complex definitions or require drawing finer distinctions. Examples may include comparing strengths and weaknesses in legal arguments about specific fact situations or flagging risks inherent in certain contractual language. A more complex approach may be necessary for using GPT-based models where longer more complex documents are involved.

The fact that OpenAI's GPT models are constantly changing presents another limitation of this work. The models we used may not be available in the future. This well-known limitation affects research with GPT models generally.

## 8 Conclusions

We evaluated several OpenAI's GPT models on three semantic annotation tasks using three corpora with diverse types of legal documents—adjudicatory opinions, contractual clauses, and statutory and regulatory provisions. We utilized the models in the zero-shot settings. The models were provided with a list of compact, one sentence long, definitions of the semantic types. The task was to assign a batch of short text snippets one of the defined categories. The results of the experiment are very promising, where the most successful GPT-4 model achieved (micro) F_1_ = 0.82 for the rhetorical roles of sentences from adjudicatory decisions, 0.90 for the types of contractual clauses, and 0.54 for the purpose of public-health system's emergency response and preparedness statutory and regulatory provisions.

We compared the approach of batch annotation to annotating one data point at a time in terms of the accuracy of the predictions as well as their cost. While analyzing one data point (i.e., text snippet) at a time yields slightly improved performance, the improvements are offset by much higher cost of performing the analysis (more than 10×). Our findings are important for legal professionals, educators and scholars who intend to leverage the capabilities of state-of-the-art LLMs to lower the cost of existing high-volume workloads, involving semantic annotation of legal documents, or to unlock novel workflows that would have not been economically feasible if performed manually or using supervised ML. The zero-shot capabilities of these LLMs appear to have potential to democratize access to the sophisticated work that traditionally has been reserved for only a small group of elite legal operations and their clients, at least for the kinds of legal tasks addressed here.

## 9 Future work

While our study of LLMs' performance on semantic annotation of short text snippets coming from diverse types of legal documents yielded valuable insights, it is subject to limitations (Section 7) and leaves much room for improvement. Hence, we suggest several directions for future work:

- Augmenting the semantic type definitions with examples should result in improved performance. This warrants further investigation.
- When employing batch prediction, that is, analyzing multiple text snippets in a single prompt, the ordering of the snippets may be of importance. In our study, we use random ordering. Understanding the effects of utilizing a particular kind of ordering, for example, the one in which the snippets appear in a document, could yield interesting insights.
- Label imbalance and/or nuanced definitions of semantic types, such as those encountered in the PHASYS data set, seem to present a formidable challenge for the zero-shot LLMs to semantically annotate legal documents.
- We also envision that the approach could be successfully combined with high-speed similarity annotation frameworks (Westermann et al., 2019, 2020) to enable highly cost efficient annotation in situations where resources are scarce.

## Data availability statement

The BVA dataset is available online at https://github.com/LLTLab/VetClaims-JSON (accessed February 9, 2023). The CUAD dataset is available online at https://www.atticusprojectai.org/cuad (accessed February 9, 2023). For information concerning the availability of the PHASYS dataset, contact information may be found at https://www.phasys.pitt.edu/contact.html. The data generated in this study are predicted labels for the data points. Requests to access the datasets should be directed to KA, ashley@pitt.edu, or JS, jsavelka@cs.cmu.edu.

## Author contributions

JS: Writing—original draft, Writing—review & editing. KA: Writing—review & editing.

## Footnotes

^1^Transformer language models are pre-trained on large text corpora. Fine-tuning the language model involves training it on new data to improve its performance on a specific task.

^2^OpenAI: ChatGPT. Available online at: https://chat.openai.com/ (accessed February 11, 2023).

^3^https://github.com/jsavelka/unreasonable_effectiveness.

^4^U.S. Department of Veterans' Appeals: Board of Veteran's Appeals. Available online at: https://www.bva.va.gov/ (accessed February 9, 2023).

^5^GitHub: VetClaims–JSON. Available online at: https://github.com/LLTLab/VetClaims-JSON (accessed February 9, 2023).

^6^Available online at: https://github.com/LLTLab/VetClaims-JSON/tree/master/LLT%20Annotation%20Protocols (accessed February 9, 2023).

^7^ https://github.com/LLTLab/VetClaims-JSON/blob/master/README.md

^8^The Atticus Project: Contract Understanding Atticus Dataset (CUAD). Available online at: https://www.atticusprojectai.org/cuad (accessed February 9, 2023).

^9^The Atticus Project: Atticus Commercial Contract Labels. Available online at: https://www.atticusprojectai.org/atticus-labels (accessed February 9, 2023).

^10^PHASYS ARM 2—LEIP Code Book. Available online at: https://www.phasys.pitt.edu/pdf/Code_Book_Numerical_Defintions.pdf (accessed February 9, 2023).

^11^Scikit learn: Random Forest Classifier. Available online at: https://scikit-learn.org/stable/modules/generated/sklearn.ensemble.RandomForestClassifier.html (accessed February 5, 2023)

^12^ github.com/pytorch/fairseq/tree/master/examples/roberta

^13^OpenAI: Model index for researchers. Available online at: https://beta.openai.com/docs/model-index-for-researchers/instructgpt-models (accessed January 15, 2023).

^14^Scikit learn: GridSearchCV. Available online at: https://scikit-learn.org/stable/modules/generated/sklearn.model_selection.GridSearchCV.html (accessed February 5, 2023). Grid search is an automated way to search for optimal values of various parameters, known as hyperparameters, that define the ML model architecture.

^15^In each epoch, the model cycles through all the training data one time.

^16^GitHub: OpenAI Python Library. Available online at: https://github.com/openai/openai-python (accessed February 9, 2023).

^17^A wrapper encloses a software application to make data compatible or abstract away complexity.

^18^Precision is the ratio of the number of positive predictions that are correct over the total number of positive predictions. Recall is the ratio of positive predictions that are correct over the number of instances that were positive. F1 is the harmonic mean of precision and recall where both measures are treated as equally important.

^19^OpenAI: Pricing. Available online at: https://openai.com/api/pricing/ (accessed February 11, 2023).

^20^The number of tokens is computed as the count of analyzed text snippets times the size of the prompt (4,097), divided by the batch size (50).
